# Supplementary material for: EnzML: multi-label prediction of enzyme classes using InterPro signatures
Source: BMC Bioinformatics. 2012 Apr 25;13:61. doi: 10.1186/1471-2105-13-61 (PMC3483700; doi:10.1186/1471-2105-13-61)
Supplement: Addtional file 5 — The Java code to format the data files, evaluate and predict. The file enzml_java_code.tar.gz contains the Java code used to format database data to ARFF and XML formats, to execute cross and train-test (jackknife) evaluations and to record evaluation results to database. More information is included in the readme.txt file and the Javadoc files. The code can be used with a MySQL database. To use a different database software, other JDBC drivers might be required. [file 1471-2105-13-61-S5.gz › java_code/utils/doc/uk/ac/ed/inf/utils/diff/Difference.html]

Difference


---


|  |  |  |
| --- | --- | --- |
| **Overview** \| **Package** \| **Class** \| **Use** \| **Tree** \| **Deprecated** \| **Index** \| **Help** | |  |
| **PREV CLASS**   NEXT CLASS | **FRAMES**    **NO FRAMES**     **All Classes** |  |
| SUMMARY: NESTED \| FIELD \| CONSTR \| METHOD | DETAIL: FIELD \| CONSTR \| METHOD |  |


---


## uk.ac.ed.inf.utils.diff Class Difference

```
java.lang.Object
  uk.ac.ed.inf.utils.diff.Difference
```

---

``` public class Difference extends java.lang.Object ```

Represents a difference, as used in `Diff`. A difference consists
of two pairs of starting and ending points, each pair representing either the
"from" or the "to" collection passed to `Diff`. If an ending point
is -1, then the difference was either a deletion or an addition. For example,
if `getDeletedEnd()` returns -1, then the difference represents an
addition.

---

| **Field Summary** | |
| --- | --- |
| `static int` | `NONE` |


**Constructor Summary**

`Difference(int delStart,
int delEnd,
int addStart,
int addEnd)`
  
          Creates the difference for the given start and end points for the
deletion and addition.


| **Method Summary** | |
| --- | --- |
| `boolean` | `equals(java.lang.Object obj)`             Compares this object to the other for equality. |
| `int` | `getAddedEnd()`             The point at which the addition ends, if any. |
| `int` | `getAddedStart()`             The point at which the addition starts, if any. |
| `int` | `getDeletedEnd()`             The point at which the deletion ends, if any. |
| `int` | `getDeletedStart()`             The point at which the deletion starts, if any. |
| `void` | `setAdded(int line)`             Sets the point as added. |
| `void` | `setDeleted(int line)`             Sets the point as deleted. |
| `java.lang.String` | `toString()`             Returns a string representation of this difference. |

**Methods inherited from class java.lang.Object**

`getClass, hashCode, notify, notifyAll, wait, wait, wait`

**Field Detail**

### NONE

```
public static final int NONE
```

**See Also:**: Constant Field Values


**Constructor Detail**

### Difference

```
public Difference(int delStart,
                  int delEnd,
                  int addStart,
                  int addEnd)
```

:   Creates the difference for the given start and end points for the
    deletion and addition.


**Method Detail**

### equals

```
public boolean equals(java.lang.Object obj)
```

:   Compares this object to the other for equality. Both objects must be of
    type Difference, with the same starting and ending points.

    :   **Overrides:**: `equals` in class `java.lang.Object`

---


### getAddedEnd

```
public int getAddedEnd()
```

:   The point at which the addition ends, if any. A value equal to
    `NONE` means this must be an addition.

---


### getAddedStart

```
public int getAddedStart()
```

:   The point at which the addition starts, if any. A value equal to
    `NONE` means this must be an addition.

---


### getDeletedEnd

```
public int getDeletedEnd()
```

:   The point at which the deletion ends, if any. A value equal to
    `NONE` means this is an addition.

---


### getDeletedStart

```
public int getDeletedStart()
```

:   The point at which the deletion starts, if any. A value equal to
    `NONE` means this is an addition.

---


### setAdded

```
public void setAdded(int line)
```

:   Sets the point as added. The start and end points will be modified to
    include the given line.

---


### setDeleted

```
public void setDeleted(int line)
```

:   Sets the point as deleted. The start and end points will be modified to
    include the given line.

---


### toString

```
public java.lang.String toString()
```

:   Returns a string representation of this difference.

    :   **Overrides:**: `toString` in class `java.lang.Object`


---


|  |  |  |
| --- | --- | --- |
| **Overview** \| **Package** \| **Class** \| **Use** \| **Tree** \| **Deprecated** \| **Index** \| **Help** | |  |
| **PREV CLASS**   NEXT CLASS | **FRAMES**    **NO FRAMES**     **All Classes** |  |
| SUMMARY: NESTED \| FIELD \| CONSTR \| METHOD | DETAIL: FIELD \| CONSTR \| METHOD |  |


---
